# Supplementary material for: MetaRibo-Seq measures translation in microbiomes
Source: Nat Commun. 2020 Jun 29;11:3268. doi: 10.1038/s41467-020-17081-z (PMC7324362; doi:10.1038/s41467-020-17081-z)
Supplement: Supplementary file 10 — Supplementary Data 7 [file 41467_2020_17081_MOESM10_ESM.zip › File2/Confidence_High_Taxonomy/222254_out.krona.html]

Javascript must be enabled to view this page.

members
magnitude
magnitudeUnassigned
count
unassigned
taxon
rank

222254\_out

9

2
9
superkingdom

976
phylum
9

9
class
200643

order
9
171549

171552
family
9

9
genus
838


SRS015794\_contig\_number\_contig-100\_191.90025SRS017307\_contig\_number\_14404SRS017307\_contig\_number\_contig-100\_1899.134651
165179
3
species

1262925

SRS013521\_contig\_number\_7279SRS049959\_contig\_number\_contig-100\_1617.270103SRS049995\_contig\_number\_487SRS053356\_contig\_number\_46907SRS078176\_contig\_number\_contig-100\_2285.102903SRS144537\_contig\_number\_contig-100\_2685.253007
6
species
